# Poly[(*N*,*N*-dimethyl­formamide-κ*O*)tris­(μ-naphthalene-1-acetato)terbium(III)]

**DOI:** 10.1107/S1600536808036155

**Published:** 2008-11-08

**Authors:** Hai-Tao Xia, Yu-Fen Liu, Ying-Ying Zhang, Da-Qi Wang

**Affiliations:** a School of Chemical Engineering, Huaihai Institute of Technology, Lianyungang 222005, People’s Republic of China; b Beilun Entry-Exit Inspection and Quarantine Bureau of China, Ningbo, Zhejiang, People’s Republic of China; c College of Chemistry and Chemical Engineering, Liaocheng University, Shandong, 252059, People’s Republic of China

## Abstract

In title compound, [Tb(C_12_H_9_O_2_)_3_(C_3_H_7_NO)]_*n*_, the Tb atom is nine-coordinated by nine O atoms from three naphthalene-1-acetate and one *N,N*-dimethyl­formamide ligands. The Tb atoms are linked by three bridging naphthalene-1-acetate ligands into a chain parallel to the *b* axis. Further stabilization of the structure is accomplished by non-classical C—H⋯O hydrogen bonds and C—H⋯π interactions.

## Related literature

For related structures, see: Xia *et al.* (2007*a* ▶,*b* ▶). 
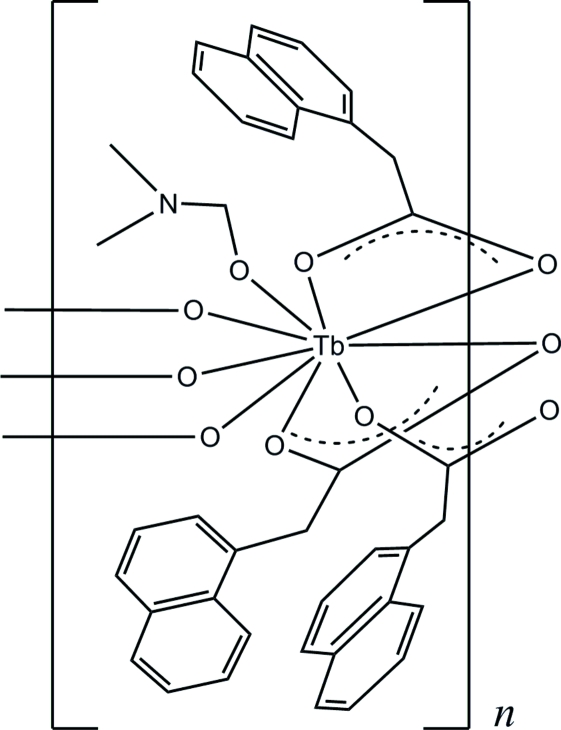


## Experimental

### 

#### Crystal data

[Tb(C_12_H_9_O_2_)_3_(C_3_H_7_NO)]
*M* *_r_* = 787.59
Monoclinic, 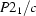

*a* = 17.6484 (18) Å
*b* = 7.8854 (10) Å
*c* = 24.184 (3) Å
β = 104.613 (2)°
*V* = 3256.7 (6) Å^3^
*Z* = 4
Mo *K*α radiation
μ = 2.23 mm^−1^
*T* = 298 (2) K
0.32 × 0.17 × 0.10 mm

#### Data collection

Bruker SMART 1000 CCD area-detector diffractometer
Absorption correction: multi-scan (*SADABS*; Sheldrick, 1996 ▶) *T*_min_ = 0.536, *T*_max_ = 0.808
15719 measured reflections
5733 independent reflections
4274 reflections with *I* > 2σ(*I*)
*R*_int_ = 0.049

#### Refinement

*R*[*F* ^2^ > 2σ(*F* ^2^)] = 0.041
*wR*(*F* ^2^) = 0.106
*S* = 1.02
5733 reflections
435 parameters
H-atom parameters constrained
Δρ_max_ = 1.17 e Å^−3^
Δρ_min_ = −1.59 e Å^−3^

### 

Data collection: *SMART* (Siemens, 1996 ▶); cell refinement: *SAINT* (Siemens, 1996 ▶); data reduction: *SAINT*; program(s) used to solve structure: *SHELXS97* (Sheldrick, 2008 ▶); program(s) used to refine structure: *SHELXL97* (Sheldrick, 2008 ▶); molecular graphics: *SHELXTL* (Sheldrick, 2008 ▶); software used to prepare material for publication: *SHELXTL*.

## Supplementary Material

Crystal structure: contains datablocks I, New_Global_Publ_Block. DOI: 10.1107/S1600536808036155/at2670sup1.cif

Structure factors: contains datablocks I. DOI: 10.1107/S1600536808036155/at2670Isup2.hkl

Additional supplementary materials:  crystallographic information; 3D view; checkCIF report

## Figures and Tables

**Table 1:** Selected bond lengths (Å)

Tb1—O4^i^	2.322 (4)
Tb1—O3	2.341 (4)
Tb1—O1^i^	2.348 (4)
Tb1—O2	2.407 (4)
Tb1—O7	2.427 (4)
Tb1—O5^i^	2.473 (4)
Tb1—O5	2.474 (4)
Tb1—O6	2.542 (4)
Tb1—O1	2.677 (4)

Symmetry code: (i) 
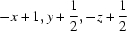
.

**Table 2:** Hydrogen-bond geometry (Å, °)

*D*—H⋯*A*	*D*—H	H⋯*A*	*D*⋯*A*	*D*—H⋯*A*
C37—H37⋯O6	0.93	2.58	3.098 (8)	116
C38—H38*A*⋯O7	0.96	2.30	2.718 (9)	105
C17—H17⋯*Cg*1^ii^	0.93	2.81	3.534 (9)	135
C39—H39*A*⋯*Cg*2^i^	0.96	2.93	3.670 (10)	135

Symmetry codes: (i) 
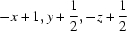
; (ii) 
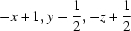
. *Cg*1 and *Cg*2 are the centroids of the C31–C36 and C15–C20 rings, respectively.

**Fig. 1.**
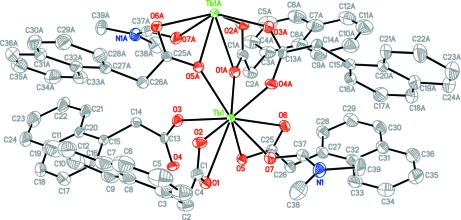
The molecular structure of (I), showing the atom-labelling scheme. Displacement ellipsoids are at the 30% probability level, H atoms have been omitted. [Symmetry codes: (A) 1 - x, 1/2 + y, 1/2 - z].

## supplementary crystallographic information

### Comment

As part of our ongoing research into the complexes between rare earth elements and naphthalene-1-acetato(NNA) and 1,10-phenanthroline(phen) ligands, we have recently reported the crystal structures of two complexes [Tb_2_(C_12_H_9_O_2_)_6_(C_12_H_8_N_2_)_2_].2C_3_H_7_NO (II) (Xia *et al.*, 2007a) and [SmTb(C_12_H_9_O_2_)_6_(C_12_H_8_N_2_)_2_].2C_3_H_7_NO (III) (Xia *et al.*, 2007b). We report here the crystal structures of a new rare earth complex with NAA, (I).

In the title complex (I), the coordination environment of the Tb atom and coordination modes of the NNA ligands coordinated to Tb^III^ ion is in agreement with the complexes reported above (Fig. 1). The average bond lengths of between the terbium center and carboxylic oxygen atoms are 2.448 (4) Å, longer than those [2.426 (7) Å and 2.440 (5) Å] of complex (II) and (III), respectively. The dihedral angles between naphthyl ring (C3–C12 ring) and another naphthyl rings are 62.69 (12)°(C15–C24 ring) and 56.17 (12)° (C27–C36 ring).

In (I), Tb atoms are linked by three naphthalene-1-acetato into a chain parallel to the *b* axis. Neighbouring chains are linked into a three-dimensional network by van Waals forces.

### Experimental

To a stirred solution of 1-naphthylacetic acid (0.5586 g, 3 mmol) in 30 ml methanol, and a solution of Tb(NO_3_)_3_.6H_2_O (0.453 g, 1.0 mmol) in water (10 ml) was added. The mixed solution was heated to 333 K and stirred for 3 h, and then cooled to room temperature. The precipitate was washed with water and then dissolved in DMF. A colourless crystal suitable for X-ray diffraction was obtained by evaporation of DMF solution.

### Refinement

All H atoms were located in difference Fourier maps. H atoms bonded to C atoms were treated as riding atoms, with C—H distances of 0.93 Å (aromatic formyl), 0.97 Å (methylene) and 0.96 Å (methyl) and *U*_iso_(H) = 1.2 (aromatic formyl methylene) or 1.5 *U*_eq_(C) (methyl).

### Crystal data

**Table:**

[Tb(C_12_H_9_O_2_)_3_(C_3_H_7_NO)]	*F*_000_ = 1584
*M**_r_* = 787.59	*D*_x_ = 1.606 Mg m^−^^3^
Monoclinic, *P*2_1_/*c*	Mo *K*α radiation λ = 0.71073 Å
Hall symbol: -P 2ybc	Cell parameters from 5059 reflections
*a* = 17.6484 (18) Å	θ = 2.4–27.5º
*b* = 7.8854 (10) Å	µ = 2.23 mm^−^^1^
*c* = 24.184 (3) Å	*T* = 298 (2) K
β = 104.613 (2)º	Block, colourless
*V* = 3256.7 (6) Å^3^	0.32 × 0.17 × 0.10 mm
*Z* = 4	

### Data collection

**Table:**

Bruker SMART 1000 CCD area-detector diffractometer	5733 independent reflections
Radiation source: fine-focus sealed tube	4274 reflections with *I* > 2σ(*I*)
Monochromator: graphite	*R*_int_ = 0.049
*T* = 298(2) K	θ_max_ = 25.0º
φ and ω scans	θ_min_ = 1.2º
Absorption correction: multi-scan(SADABS; Sheldrick, 1996)	*h* = −20→17
*T*_min_ = 0.536, *T*_max_ = 0.808	*k* = −9→9
15719 measured reflections	*l* = −28→28

### Refinement

**Table:**

Refinement on *F*^2^	Secondary atom site location: difference Fourier map
Least-squares matrix: full	Hydrogen site location: inferred from neighbouring sites
*R*[*F*^2^ > 2σ(*F*^2^)] = 0.041	H-atom parameters constrained
*wR*(*F*^2^) = 0.106	*w* = 1/[σ^2^(*F*_o_^2^) + (0.0533*P*)^2^ + 2.7161*P*] where *P* = (*F*_o_^2^ + 2*F*_c_^2^)/3
*S* = 1.02	(Δ/σ)_max_ = 0.001
5733 reflections	Δρ_max_ = 1.17 e Å^−^^3^
435 parameters	Δρ_min_ = −1.59 e Å^−^^3^
Primary atom site location: structure-invariant direct methods	Extinction correction: none

### Special details

**Table:**

Geometry. All e.s.d.'s (except the e.s.d. in the dihedral angle between two l.s. planes) are estimated using the full covariance matrix. The cell e.s.d.'s are taken into account individually in the estimation of e.s.d.'s in distances, angles and torsion angles; correlations between e.s.d.'s in cell parameters are only used when they are defined by crystal symmetry. An approximate (isotropic) treatment of cell e.s.d.'s is used for estimating e.s.d.'s involving l.s. planes.
Refinement. Refinement of *F*^2^ against ALL reflections. The weighted *R*-factor *wR* and goodness of fit *S* are based on *F*^2^, conventional *R*-factors *R* are based on *F*, with *F* set to zero for negative *F*^2^. The threshold expression of *F*^2^ > σ(*F*^2^) is used only for calculating *R*-factors(gt) *etc*. and is not relevant to the choice of reflections for refinement. *R*-factors based on *F*^2^ are statistically about twice as large as those based on *F*, and *R*- factors based on ALL data will be even larger.

### Fractional atomic coordinates and isotropic or equivalent isotropic displacement parameters (Å^2^)

**Table:**

	*x*	*y*	*z*	*U*_iso_*/*U*_eq_	
Tb1	0.524810 (16)	0.42775 (4)	0.272830 (11)	0.02364 (11)	
N1	0.7253 (3)	0.2677 (8)	0.4209 (2)	0.0470 (15)	
O1	0.4761 (2)	0.1248 (5)	0.30008 (18)	0.0339 (10)	
O2	0.4655 (3)	0.3527 (6)	0.34879 (18)	0.0390 (11)	
O3	0.3993 (2)	0.3832 (6)	0.21354 (18)	0.0380 (11)	
O4	0.3868 (2)	0.1228 (5)	0.17513 (18)	0.0348 (11)	
O5	0.5499 (2)	0.1847 (5)	0.21524 (16)	0.0309 (10)	
O6	0.6464 (2)	0.3687 (6)	0.23763 (19)	0.0381 (11)	
O7	0.6182 (3)	0.2771 (6)	0.34752 (19)	0.0437 (12)	
C1	0.4615 (4)	0.1964 (9)	0.3431 (3)	0.0354 (16)	
C2	0.4441 (5)	0.0852 (9)	0.3892 (3)	0.0484 (19)	
H2A	0.4927	0.0359	0.4112	0.058*	
H2B	0.4103	−0.0069	0.3713	0.058*	
C3	0.4052 (5)	0.1789 (10)	0.4294 (3)	0.056 (2)	
C4	0.4480 (6)	0.2148 (11)	0.4841 (3)	0.067 (2)	
H4	0.4998	0.1797	0.4967	0.080*	
C5	0.4107 (7)	0.3091 (13)	0.5221 (4)	0.080 (3)	
H5	0.4382	0.3365	0.5592	0.096*	
C6	0.3359 (7)	0.3546 (13)	0.5022 (5)	0.081 (3)	
H6	0.3129	0.4137	0.5271	0.098*	
C7	0.2894 (7)	0.3223 (11)	0.4482 (5)	0.072 (3)	
C8	0.3263 (6)	0.2311 (10)	0.4100 (4)	0.059 (2)	
C9	0.2801 (6)	0.2008 (11)	0.3545 (4)	0.066 (2)	
H9	0.3017	0.1398	0.3293	0.079*	
C10	0.2047 (6)	0.2572 (12)	0.3361 (5)	0.080 (3)	
H10	0.1768	0.2388	0.2985	0.096*	
C11	0.1701 (7)	0.3416 (14)	0.3733 (6)	0.088 (3)	
H11	0.1183	0.3770	0.3609	0.105*	
C12	0.2101 (7)	0.3724 (12)	0.4265 (5)	0.076 (3)	
H12	0.1853	0.4293	0.4506	0.091*	
C13	0.3652 (3)	0.2728 (9)	0.1786 (3)	0.0301 (15)	
C14	0.2921 (4)	0.3284 (9)	0.1342 (3)	0.0366 (16)	
H14A	0.2846	0.4488	0.1390	0.044*	
H14B	0.3009	0.3120	0.0966	0.044*	
C15	0.2176 (4)	0.2374 (8)	0.1364 (3)	0.0357 (16)	
C16	0.2114 (4)	0.1483 (9)	0.1830 (3)	0.0458 (18)	
H16	0.2549	0.1389	0.2139	0.055*	
C17	0.1408 (5)	0.0696 (10)	0.1858 (4)	0.063 (2)	
H17	0.1381	0.0069	0.2179	0.075*	
C18	0.0766 (5)	0.0864 (11)	0.1410 (4)	0.063 (2)	
H18	0.0296	0.0376	0.1436	0.075*	
C19	0.0788 (4)	0.1738 (11)	0.0917 (4)	0.056 (2)	
C20	0.1505 (4)	0.2507 (9)	0.0887 (3)	0.0427 (18)	
C21	0.1516 (4)	0.3357 (10)	0.0381 (3)	0.051 (2)	
H21	0.1980	0.3864	0.0352	0.061*	
C22	0.0872 (5)	0.3475 (12)	−0.0073 (4)	0.066 (2)	
H22	0.0896	0.4057	−0.0403	0.079*	
C23	0.0173 (5)	0.2690 (12)	−0.0027 (4)	0.077 (3)	
H23	−0.0268	0.2749	−0.0333	0.092*	
C24	0.0130 (5)	0.1872 (12)	0.0440 (4)	0.069 (3)	
H24	−0.0341	0.1373	0.0456	0.082*	
C25	0.6191 (4)	0.2342 (8)	0.2145 (3)	0.0319 (15)	
C26	0.6633 (4)	0.1225 (9)	0.1826 (3)	0.0477 (19)	
H26A	0.6296	0.0998	0.1449	0.057*	
H26B	0.6737	0.0149	0.2025	0.057*	
C27	0.7401 (4)	0.1926 (9)	0.1754 (3)	0.0455 (19)	
C28	0.7411 (5)	0.2700 (10)	0.1254 (4)	0.053 (2)	
H28	0.6943	0.2827	0.0976	0.063*	
C29	0.8106 (5)	0.3315 (11)	0.1143 (4)	0.061 (2)	
H29	0.8092	0.3841	0.0796	0.073*	
C30	0.8794 (5)	0.3141 (10)	0.1539 (4)	0.059 (2)	
H30	0.9253	0.3526	0.1458	0.071*	
C31	0.8830 (4)	0.2387 (10)	0.2073 (4)	0.052 (2)	
C32	0.8112 (4)	0.1781 (9)	0.2182 (3)	0.0488 (19)	
C33	0.8159 (5)	0.1038 (10)	0.2716 (4)	0.058 (2)	
H33	0.7704	0.0641	0.2800	0.069*	
C34	0.8852 (6)	0.0884 (11)	0.3115 (4)	0.072 (3)	
H34	0.8865	0.0377	0.3464	0.087*	
C35	0.9549 (6)	0.1483 (12)	0.3005 (5)	0.078 (3)	
H35	1.0021	0.1380	0.3280	0.094*	
C36	0.9526 (5)	0.2211 (12)	0.2494 (4)	0.068 (2)	
H36	0.9989	0.2605	0.2423	0.081*	
C37	0.6886 (4)	0.3035 (9)	0.3681 (3)	0.0412 (17)	
H37	0.7173	0.3518	0.3448	0.049*	
C38	0.6820 (5)	0.2000 (12)	0.4591 (3)	0.067 (2)	
H38A	0.6270	0.2052	0.4412	0.101*	
H38B	0.6932	0.2654	0.4937	0.101*	
H38C	0.6971	0.0841	0.4679	0.101*	
C39	0.8097 (4)	0.2947 (11)	0.4423 (4)	0.067 (2)	
H39A	0.8311	0.3311	0.4115	0.100*	
H39B	0.8343	0.1906	0.4579	0.100*	
H39C	0.8191	0.3801	0.4715	0.100*	

### Atomic displacement parameters (Å^2^)

**Table:**

	*U*^11^	*U*^22^	*U*^33^	*U*^12^	*U*^13^	*U*^23^
Tb1	0.02147 (16)	0.02162 (17)	0.02753 (16)	−0.00010 (14)	0.00560 (11)	−0.00052 (14)
N1	0.039 (4)	0.049 (4)	0.046 (4)	0.000 (3)	−0.001 (3)	−0.005 (3)
O1	0.035 (3)	0.035 (3)	0.034 (2)	0.000 (2)	0.013 (2)	−0.002 (2)
O2	0.050 (3)	0.031 (3)	0.042 (3)	0.005 (2)	0.024 (2)	0.006 (2)
O3	0.029 (3)	0.037 (3)	0.042 (3)	0.001 (2)	−0.003 (2)	−0.006 (2)
O4	0.040 (3)	0.023 (3)	0.038 (3)	0.005 (2)	0.003 (2)	0.0023 (19)
O5	0.030 (2)	0.029 (3)	0.037 (2)	−0.0011 (19)	0.016 (2)	−0.003 (2)
O6	0.032 (3)	0.032 (3)	0.054 (3)	−0.005 (2)	0.018 (2)	−0.010 (2)
O7	0.034 (3)	0.038 (3)	0.054 (3)	−0.002 (2)	0.002 (2)	0.010 (2)
C1	0.038 (4)	0.034 (4)	0.039 (4)	0.003 (3)	0.017 (3)	0.004 (3)
C2	0.069 (5)	0.039 (4)	0.047 (4)	0.003 (4)	0.031 (4)	0.005 (4)
C3	0.084 (6)	0.041 (5)	0.056 (5)	−0.004 (4)	0.040 (5)	0.011 (4)
C4	0.097 (7)	0.053 (6)	0.060 (6)	0.000 (5)	0.039 (5)	0.004 (4)
C5	0.125 (10)	0.068 (7)	0.056 (6)	−0.007 (7)	0.040 (6)	0.003 (5)
C6	0.114 (9)	0.057 (6)	0.089 (8)	0.005 (6)	0.057 (7)	0.007 (6)
C7	0.098 (8)	0.047 (6)	0.087 (7)	−0.003 (5)	0.057 (6)	0.015 (5)
C8	0.082 (7)	0.040 (5)	0.073 (6)	−0.003 (4)	0.051 (5)	0.011 (4)
C9	0.079 (7)	0.049 (6)	0.082 (7)	0.000 (5)	0.041 (6)	0.011 (5)
C10	0.085 (8)	0.058 (6)	0.105 (8)	0.000 (6)	0.038 (6)	0.010 (6)
C11	0.089 (8)	0.065 (7)	0.121 (10)	0.001 (6)	0.049 (8)	0.018 (7)
C12	0.091 (8)	0.055 (6)	0.103 (8)	0.007 (5)	0.064 (7)	0.009 (6)
C13	0.020 (3)	0.036 (4)	0.034 (4)	−0.002 (3)	0.006 (3)	0.007 (3)
C14	0.032 (4)	0.029 (4)	0.044 (4)	−0.002 (3)	0.002 (3)	0.006 (3)
C15	0.029 (4)	0.028 (4)	0.050 (4)	0.003 (3)	0.012 (3)	0.000 (3)
C16	0.037 (4)	0.041 (4)	0.061 (5)	0.006 (3)	0.015 (4)	0.004 (4)
C17	0.056 (6)	0.051 (5)	0.087 (6)	−0.005 (4)	0.031 (5)	0.008 (5)
C18	0.038 (5)	0.055 (6)	0.098 (7)	−0.003 (4)	0.021 (5)	−0.004 (5)
C19	0.034 (5)	0.050 (5)	0.081 (6)	0.004 (4)	0.010 (4)	−0.012 (5)
C20	0.030 (4)	0.033 (4)	0.064 (5)	0.003 (3)	0.009 (3)	−0.009 (4)
C21	0.032 (4)	0.050 (5)	0.062 (5)	0.004 (4)	−0.004 (4)	−0.005 (4)
C22	0.046 (5)	0.074 (6)	0.068 (6)	0.010 (5)	−0.003 (4)	−0.003 (5)
C23	0.049 (6)	0.076 (7)	0.090 (7)	0.004 (5)	−0.012 (5)	−0.013 (6)
C24	0.038 (5)	0.063 (6)	0.097 (7)	−0.001 (4)	0.001 (5)	−0.009 (6)
C25	0.032 (4)	0.026 (4)	0.043 (4)	0.001 (3)	0.019 (3)	0.001 (3)
C26	0.040 (4)	0.038 (4)	0.073 (5)	−0.004 (3)	0.029 (4)	−0.015 (4)
C27	0.042 (4)	0.035 (4)	0.067 (5)	−0.005 (3)	0.029 (4)	−0.016 (4)
C28	0.052 (5)	0.049 (5)	0.066 (5)	−0.004 (4)	0.029 (4)	−0.011 (4)
C29	0.068 (6)	0.053 (6)	0.070 (6)	−0.007 (5)	0.034 (5)	−0.011 (5)
C30	0.057 (6)	0.052 (6)	0.081 (6)	−0.013 (4)	0.042 (5)	−0.014 (5)
C31	0.044 (5)	0.040 (5)	0.080 (6)	−0.003 (4)	0.030 (4)	−0.014 (4)
C32	0.048 (5)	0.033 (4)	0.073 (5)	−0.001 (4)	0.029 (4)	−0.011 (4)
C33	0.058 (6)	0.040 (5)	0.081 (6)	−0.002 (4)	0.028 (5)	−0.002 (4)
C34	0.079 (7)	0.050 (6)	0.087 (7)	−0.001 (5)	0.018 (6)	0.007 (5)
C35	0.064 (7)	0.059 (6)	0.105 (8)	0.007 (5)	0.009 (6)	−0.006 (6)
C36	0.055 (6)	0.055 (6)	0.096 (7)	0.000 (4)	0.025 (5)	−0.011 (5)
C37	0.038 (4)	0.031 (4)	0.052 (5)	−0.002 (3)	0.006 (4)	0.000 (3)
C38	0.070 (6)	0.082 (7)	0.047 (5)	0.015 (5)	0.008 (4)	0.002 (5)
C39	0.032 (4)	0.069 (6)	0.086 (6)	−0.002 (4)	−0.013 (4)	−0.005 (5)

### Geometric parameters (Å, °)

**Table:**

Tb1—O4^i^	2.322 (4)	C15—C16	1.357 (9)
Tb1—O3	2.341 (4)	C15—C20	1.433 (9)
Tb1—O1^i^	2.348 (4)	C16—C17	1.409 (10)
Tb1—O2	2.407 (4)	C16—H16	0.9300
Tb1—O7	2.427 (4)	C17—C18	1.363 (12)
Tb1—O5^i^	2.473 (4)	C17—H17	0.9300
Tb1—O5	2.474 (4)	C18—C19	1.387 (11)
Tb1—O6	2.542 (4)	C18—H18	0.9300
Tb1—O1	2.677 (4)	C19—C24	1.420 (10)
N1—C37	1.307 (8)	C19—C20	1.421 (10)
N1—C38	1.442 (9)	C20—C21	1.399 (10)
N1—C39	1.465 (8)	C21—C22	1.370 (10)
O1—C1	1.265 (7)	C21—H21	0.9300
O1—Tb1^ii^	2.348 (4)	C22—C23	1.410 (12)
O2—C1	1.240 (8)	C22—H22	0.9300
O3—C13	1.256 (7)	C23—C24	1.319 (12)
O4—C13	1.252 (7)	C23—H23	0.9300
O4—Tb1^ii^	2.322 (4)	C24—H24	0.9300
O5—C25	1.287 (7)	C25—C26	1.510 (9)
O5—Tb1^ii^	2.473 (4)	C26—C27	1.514 (9)
O6—C25	1.239 (7)	C26—H26A	0.9700
O7—C37	1.234 (7)	C26—H26B	0.9700
C1—C2	1.509 (9)	C27—C28	1.359 (10)
C2—C3	1.517 (10)	C27—C32	1.416 (10)
C2—H2A	0.9700	C28—C29	1.406 (10)
C2—H2B	0.9700	C28—H28	0.9300
C3—C4	1.377 (11)	C29—C30	1.351 (11)
C3—C8	1.413 (11)	C29—H29	0.9300
C4—C5	1.462 (12)	C30—C31	1.408 (11)
C4—H4	0.9300	C30—H30	0.9300
C5—C6	1.335 (13)	C31—C36	1.391 (11)
C5—H5	0.9300	C31—C32	1.439 (10)
C6—C7	1.379 (13)	C32—C33	1.403 (10)
C6—H6	0.9300	C33—C34	1.358 (12)
C7—C12	1.422 (13)	C33—H33	0.9300
C7—C8	1.449 (11)	C34—C35	1.404 (12)
C8—C9	1.404 (12)	C34—H34	0.9300
C9—C10	1.367 (12)	C35—C36	1.353 (12)
C9—H9	0.9300	C35—H35	0.9300
C10—C11	1.379 (13)	C36—H36	0.9300
C10—H10	0.9300	C37—H37	0.9300
C11—C12	1.324 (13)	C38—H38A	0.9600
C11—H11	0.9300	C38—H38B	0.9600
C12—H12	0.9300	C38—H38C	0.9600
C13—C14	1.520 (8)	C39—H39A	0.9600
C14—C15	1.509 (9)	C39—H39B	0.9600
C14—H14A	0.9700	C39—H39C	0.9600
C14—H14B	0.9700		
			
O4^i^—Tb1—O3	146.45 (15)	O3—C13—C14	116.9 (6)
O4^i^—Tb1—O1^i^	81.07 (15)	C15—C14—C13	115.5 (5)
O3—Tb1—O1^i^	79.23 (15)	C15—C14—H14A	108.4
O4^i^—Tb1—O2	96.39 (16)	C13—C14—H14A	108.4
O3—Tb1—O2	84.51 (16)	C15—C14—H14B	108.4
O1^i^—Tb1—O2	144.54 (15)	C13—C14—H14B	108.4
O4^i^—Tb1—O7	71.57 (16)	H14A—C14—H14B	107.5
O3—Tb1—O7	137.93 (16)	C16—C15—C20	118.9 (6)
O1^i^—Tb1—O7	139.27 (15)	C16—C15—C14	121.9 (6)
O2—Tb1—O7	69.87 (15)	C20—C15—C14	119.2 (6)
O4^i^—Tb1—O5^i^	72.48 (14)	C15—C16—C17	121.8 (7)
O3—Tb1—O5^i^	75.20 (14)	C15—C16—H16	119.1
O1^i^—Tb1—O5^i^	69.06 (14)	C17—C16—H16	119.1
O2—Tb1—O5^i^	76.42 (14)	C18—C17—C16	119.1 (8)
O7—Tb1—O5^i^	126.59 (15)	C18—C17—H17	120.4
O4^i^—Tb1—O5	128.86 (14)	C16—C17—H17	120.4
O3—Tb1—O5	79.33 (14)	C17—C18—C19	122.3 (8)
O1^i^—Tb1—O5	93.34 (14)	C17—C18—H18	118.9
O2—Tb1—O5	114.40 (15)	C19—C18—H18	118.9
O7—Tb1—O5	81.68 (15)	C18—C19—C24	122.8 (8)
O5^i^—Tb1—O5	151.24 (3)	C18—C19—C20	118.4 (7)
O4^i^—Tb1—O6	78.16 (15)	C24—C19—C20	118.9 (8)
O3—Tb1—O6	121.00 (15)	C21—C20—C19	117.3 (7)
O1^i^—Tb1—O6	73.50 (14)	C21—C20—C15	123.2 (6)
O2—Tb1—O6	140.97 (15)	C19—C20—C15	119.6 (7)
O7—Tb1—O6	71.80 (15)	C22—C21—C20	122.8 (8)
O5^i^—Tb1—O6	135.12 (14)	C22—C21—H21	118.6
O5—Tb1—O6	51.96 (13)	C20—C21—H21	118.6
O4^i^—Tb1—O1	132.16 (14)	C21—C22—C23	118.2 (9)
O3—Tb1—O1	72.78 (14)	C21—C22—H22	120.9
O1^i^—Tb1—O1	146.51 (8)	C23—C22—H22	120.9
O2—Tb1—O1	50.50 (14)	C24—C23—C22	121.6 (9)
O7—Tb1—O1	65.16 (14)	C24—C23—H23	119.2
O5^i^—Tb1—O1	119.23 (13)	C22—C23—H23	119.2
O5—Tb1—O1	63.99 (13)	C23—C24—C19	121.3 (9)
O6—Tb1—O1	105.65 (13)	C23—C24—H24	119.3
C37—N1—C38	119.6 (6)	C19—C24—H24	119.3
C37—N1—C39	121.6 (7)	O6—C25—O5	121.0 (6)
C38—N1—C39	118.9 (6)	O6—C25—C26	122.6 (6)
C1—O1—Tb1^ii^	161.5 (4)	O5—C25—C26	116.4 (6)
C1—O1—Tb1	87.1 (4)	C25—C26—C27	116.0 (6)
Tb1^ii^—O1—Tb1	110.33 (15)	C25—C26—H26A	108.3
C1—O2—Tb1	100.5 (4)	C27—C26—H26A	108.3
C13—O3—Tb1	137.3 (4)	C25—C26—H26B	108.3
C13—O4—Tb1^ii^	139.8 (4)	C27—C26—H26B	108.3
C25—O5—Tb1^ii^	141.8 (4)	H26A—C26—H26B	107.4
C25—O5—Tb1	94.5 (4)	C28—C27—C32	118.6 (7)
Tb1^ii^—O5—Tb1	113.12 (15)	C28—C27—C26	118.7 (7)
C25—O6—Tb1	92.5 (4)	C32—C27—C26	122.7 (7)
C37—O7—Tb1	130.1 (4)	C27—C28—C29	122.3 (8)
O2—C1—O1	121.1 (6)	C27—C28—H28	118.9
O2—C1—C2	120.7 (6)	C29—C28—H28	118.9
O1—C1—C2	118.0 (6)	C30—C29—C28	120.0 (8)
C1—C2—C3	113.6 (6)	C30—C29—H29	120.0
C1—C2—H2A	108.8	C28—C29—H29	120.0
C3—C2—H2A	108.8	C29—C30—C31	121.2 (7)
C1—C2—H2B	108.8	C29—C30—H30	119.4
C3—C2—H2B	108.8	C31—C30—H30	119.4
H2A—C2—H2B	107.7	C36—C31—C30	122.7 (8)
C4—C3—C8	121.0 (8)	C36—C31—C32	119.2 (8)
C4—C3—C2	119.3 (8)	C30—C31—C32	118.0 (8)
C8—C3—C2	119.7 (8)	C33—C32—C27	122.8 (7)
C3—C4—C5	118.9 (9)	C33—C32—C31	117.2 (8)
C3—C4—H4	120.5	C27—C32—C31	119.9 (7)
C5—C4—H4	120.5	C34—C33—C32	121.6 (8)
C6—C5—C4	118.1 (9)	C34—C33—H33	119.2
C6—C5—H5	120.9	C32—C33—H33	119.2
C4—C5—H5	120.9	C33—C34—C35	120.7 (9)
C5—C6—C7	126.1 (10)	C33—C34—H34	119.6
C5—C6—H6	117.0	C35—C34—H34	119.6
C7—C6—H6	117.0	C36—C35—C34	119.3 (9)
C6—C7—C12	126.4 (10)	C36—C35—H35	120.4
C6—C7—C8	116.1 (10)	C34—C35—H35	120.4
C12—C7—C8	117.5 (10)	C35—C36—C31	121.9 (9)
C9—C8—C3	123.7 (8)	C35—C36—H36	119.1
C9—C8—C7	116.6 (9)	C31—C36—H36	119.1
C3—C8—C7	119.7 (9)	O7—C37—N1	123.9 (7)
C10—C9—C8	122.7 (9)	O7—C37—H37	118.0
C10—C9—H9	118.7	N1—C37—H37	118.0
C8—C9—H9	118.7	N1—C38—H38A	109.5
C9—C10—C11	119.9 (11)	N1—C38—H38B	109.5
C9—C10—H10	120.0	H38A—C38—H38B	109.5
C11—C10—H10	120.0	N1—C38—H38C	109.5
C12—C11—C10	120.4 (11)	H38A—C38—H38C	109.5
C12—C11—H11	119.8	H38B—C38—H38C	109.5
C10—C11—H11	119.8	N1—C39—H39A	109.5
C11—C12—C7	122.9 (10)	N1—C39—H39B	109.5
C11—C12—H12	118.6	H39A—C39—H39B	109.5
C7—C12—H12	118.6	N1—C39—H39C	109.5
O4—C13—O3	126.7 (6)	H39A—C39—H39C	109.5
O4—C13—C14	116.3 (6)	H39B—C39—H39C	109.5
			
O4^i^—Tb1—O1—C1	51.7 (4)	C5—C6—C7—C8	−0.3 (15)
O3—Tb1—O1—C1	−102.0 (4)	C4—C3—C8—C9	178.9 (8)
O1^i^—Tb1—O1—C1	−136.6 (4)	C2—C3—C8—C9	0.3 (11)
O2—Tb1—O1—C1	−4.7 (4)	C4—C3—C8—C7	−0.6 (12)
O7—Tb1—O1—C1	79.0 (4)	C2—C3—C8—C7	−179.3 (7)
O5^i^—Tb1—O1—C1	−40.6 (4)	C6—C7—C8—C9	−178.7 (8)
O5—Tb1—O1—C1	171.7 (4)	C12—C7—C8—C9	0.2 (11)
O6—Tb1—O1—C1	139.8 (4)	C6—C7—C8—C3	0.9 (12)
O4^i^—Tb1—O1—Tb1^ii^	−134.69 (18)	C12—C7—C8—C3	179.8 (7)
O3—Tb1—O1—Tb1^ii^	71.61 (18)	C3—C8—C9—C10	−177.8 (8)
O1^i^—Tb1—O1—Tb1^ii^	37.0 (2)	C7—C8—C9—C10	1.7 (12)
O2—Tb1—O1—Tb1^ii^	168.8 (3)	C8—C9—C10—C11	−2.8 (14)
O7—Tb1—O1—Tb1^ii^	−107.5 (2)	C9—C10—C11—C12	1.8 (15)
O5^i^—Tb1—O1—Tb1^ii^	132.94 (15)	C10—C11—C12—C7	0.1 (16)
O5—Tb1—O1—Tb1^ii^	−14.70 (14)	C6—C7—C12—C11	177.6 (10)
O6—Tb1—O1—Tb1^ii^	−46.63 (19)	C8—C7—C12—C11	−1.1 (14)
O4^i^—Tb1—O2—C1	−136.6 (4)	Tb1^ii^—O4—C13—O3	13.1 (11)
O3—Tb1—O2—C1	77.1 (4)	Tb1^ii^—O4—C13—C14	−169.7 (4)
O1^i^—Tb1—O2—C1	139.8 (4)	Tb1—O3—C13—O4	19.4 (10)
O7—Tb1—O2—C1	−69.0 (4)	Tb1—O3—C13—C14	−157.8 (4)
O5^i^—Tb1—O2—C1	153.2 (4)	O4—C13—C14—C15	63.9 (8)
O5—Tb1—O2—C1	1.4 (4)	O3—C13—C14—C15	−118.6 (6)
O6—Tb1—O2—C1	−57.6 (5)	C13—C14—C15—C16	17.4 (10)
O1—Tb1—O2—C1	4.9 (4)	C13—C14—C15—C20	−165.0 (6)
O4^i^—Tb1—O3—C13	160.7 (5)	C20—C15—C16—C17	−0.3 (11)
O1^i^—Tb1—O3—C13	105.6 (6)	C14—C15—C16—C17	177.2 (7)
O2—Tb1—O3—C13	−106.0 (6)	C15—C16—C17—C18	−1.4 (12)
O7—Tb1—O3—C13	−54.5 (7)	C16—C17—C18—C19	2.2 (13)
O5^i^—Tb1—O3—C13	176.6 (6)	C17—C18—C19—C24	177.7 (8)
O5—Tb1—O3—C13	10.1 (6)	C17—C18—C19—C20	−1.2 (12)
O6—Tb1—O3—C13	42.5 (6)	C18—C19—C20—C21	179.0 (7)
O1—Tb1—O3—C13	−55.8 (6)	C24—C19—C20—C21	−0.1 (11)
O4^i^—Tb1—O5—C25	−14.3 (4)	C18—C19—C20—C15	−0.6 (11)
O3—Tb1—O5—C25	145.3 (4)	C24—C19—C20—C15	−179.6 (7)
O1^i^—Tb1—O5—C25	66.9 (4)	C16—C15—C20—C21	−178.2 (7)
O2—Tb1—O5—C25	−135.8 (3)	C14—C15—C20—C21	4.2 (10)
O7—Tb1—O5—C25	−72.4 (4)	C16—C15—C20—C19	1.3 (10)
O5^i^—Tb1—O5—C25	117.3 (3)	C14—C15—C20—C19	−176.3 (6)
O6—Tb1—O5—C25	0.9 (3)	C19—C20—C21—C22	0.3 (11)
O1—Tb1—O5—C25	−138.8 (4)	C15—C20—C21—C22	179.8 (7)
O4^i^—Tb1—O5—Tb1^ii^	138.69 (17)	C20—C21—C22—C23	−0.6 (13)
O3—Tb1—O5—Tb1^ii^	−61.71 (17)	C21—C22—C23—C24	0.6 (14)
O1^i^—Tb1—O5—Tb1^ii^	−140.09 (17)	C22—C23—C24—C19	−0.3 (15)
O2—Tb1—O5—Tb1^ii^	17.2 (2)	C18—C19—C24—C23	−178.9 (9)
O7—Tb1—O5—Tb1^ii^	80.57 (18)	C20—C19—C24—C23	0.1 (13)
O5^i^—Tb1—O5—Tb1^ii^	−89.7 (3)	Tb1—O6—C25—O5	1.7 (6)
O6—Tb1—O5—Tb1^ii^	153.9 (3)	Tb1—O6—C25—C26	179.9 (6)
O1—Tb1—O5—Tb1^ii^	14.21 (14)	Tb1^ii^—O5—C25—O6	−139.3 (5)
O4^i^—Tb1—O6—C25	166.9 (4)	Tb1—O5—C25—O6	−1.8 (6)
O3—Tb1—O6—C25	−42.9 (4)	Tb1^ii^—O5—C25—C26	42.5 (9)
O1^i^—Tb1—O6—C25	−109.0 (4)	Tb1—O5—C25—C26	179.9 (5)
O2—Tb1—O6—C25	81.4 (4)	O6—C25—C26—C27	−5.8 (11)
O7—Tb1—O6—C25	92.7 (4)	O5—C25—C26—C27	172.4 (6)
O5^i^—Tb1—O6—C25	−143.3 (4)	C25—C26—C27—C28	−97.8 (8)
O5—Tb1—O6—C25	−1.0 (3)	C25—C26—C27—C32	83.4 (9)
O1—Tb1—O6—C25	36.1 (4)	C32—C27—C28—C29	1.8 (11)
O4^i^—Tb1—O7—C37	−34.6 (6)	C26—C27—C28—C29	−177.0 (7)
O3—Tb1—O7—C37	165.0 (5)	C27—C28—C29—C30	0.2 (12)
O1^i^—Tb1—O7—C37	15.7 (7)	C28—C29—C30—C31	−1.6 (13)
O2—Tb1—O7—C37	−138.9 (6)	C29—C30—C31—C36	−179.0 (8)
O5^i^—Tb1—O7—C37	−84.7 (6)	C29—C30—C31—C32	0.9 (12)
O5—Tb1—O7—C37	101.2 (6)	C28—C27—C32—C33	178.6 (7)
O6—Tb1—O7—C37	48.6 (6)	C26—C27—C32—C33	−2.6 (11)
O1—Tb1—O7—C37	166.3 (6)	C28—C27—C32—C31	−2.5 (11)
Tb1—O2—C1—O1	−9.4 (7)	C26—C27—C32—C31	176.3 (6)
Tb1—O2—C1—C2	166.5 (5)	C36—C31—C32—C33	0.1 (11)
Tb1^ii^—O1—C1—O2	−152.4 (10)	C30—C31—C32—C33	−179.8 (7)
Tb1—O1—C1—O2	8.3 (6)	C36—C31—C32—C27	−179.0 (7)
Tb1^ii^—O1—C1—C2	31.6 (17)	C30—C31—C32—C27	1.1 (11)
Tb1—O1—C1—C2	−167.7 (6)	C27—C32—C33—C34	178.6 (8)
O2—C1—C2—C3	20.5 (10)	C31—C32—C33—C34	−0.4 (11)
O1—C1—C2—C3	−163.4 (7)	C32—C33—C34—C35	0.5 (13)
C1—C2—C3—C4	−106.7 (8)	C33—C34—C35—C36	−0.4 (14)
C1—C2—C3—C8	72.0 (9)	C34—C35—C36—C31	0.1 (14)
C8—C3—C4—C5	−0.3 (12)	C30—C31—C36—C35	180.0 (8)
C2—C3—C4—C5	178.4 (7)	C32—C31—C36—C35	0.1 (13)
C3—C4—C5—C6	0.8 (13)	Tb1—O7—C37—N1	149.8 (5)
C4—C5—C6—C7	−0.5 (16)	C38—N1—C37—O7	−2.9 (11)
C5—C6—C7—C12	−179.1 (10)	C39—N1—C37—O7	177.2 (7)

Symmetry codes: (i) −*x*+1, *y*+1/2, −*z*+1/2; (ii) −*x*+1, *y*−1/2, −*z*+1/2.

### Hydrogen-bond geometry (Å, °)

**Table:**

*D*—H···*A*	*D*—H	H···*A*	*D*···*A*	*D*—H···*A*
C37—H37···O6	0.93	2.58	3.098 (8)	116
C38—H38A···O7	0.96	2.30	2.718 (9)	105
C17—H17···Cg1^ii^	0.93	2.81	3.534 (9)	135
C39—H39A···Cg2^i^	0.96	2.93	3.670 (10)	135

Symmetry codes: (ii) −*x*+1, *y*−1/2, −*z*+1/2; (i) −*x*+1, *y*+1/2, −*z*+1/2.
